# Supplementary material for: Impaired extinction of cocaine seeking in HIV-infected mice is accompanied by peripheral and central immune dysregulation
Source: Commun Biol. 2024 Mar 30;7:387. doi: 10.1038/s42003-024-06079-8 (PMC10980811; doi:10.1038/s42003-024-06079-8)
Supplement: Supplementary file 2 — Supplementary Information [file 42003_2024_6079_MOESM2_ESM.pdf]

Supplemental Table 1. Values of Analytes detected by Mouse Cytokine/Chemokine Panel A 48-Plex Discovery Assay				
**Analyte (units)**	**Abbreviation**	**Effect of HIV**	**Effect of Cocaine**	**Interaction**
Eotaxin	Eotaxin/CCL11	F (1, 28) = 0.1284, P=0.7228	F (1, 28) = 0.07248, P=0.7897	F (1, 28) = 0.1284, P=0.7228
Granulocyte-colony stimulating factor	G-CSF	F (1, 28) = 4.723, P=0.0384	F (1, 28) = 0.2005, P=0.6577	F (1, 28) = 0.6481, P=0.4276
Interleukin 1-alpha	IL-1α	F (1, 26) = 6.210, P=0.0194	F (1, 26) = 0.0006173, P=0.9804	F (1, 26) = 0.01181, P=0.9143
Interleukin 6	IL-6	F (1, 28) = 1.059, P=0.3123	F (1, 28) = 1.656, P=0.2087	F (1, 28) = 0.9972, P=0.3265
Interleukin 12 subunit	IL-12p40	F (1, 23) = 1.075, P=0.3106	F (1, 23) = 1.270, P=0.2714	F (1, 23) = 0.003452, P=0.9537
Interferon gamma inducible protein-10	IP-10	F (1, 28) = 5.094, P=0.0320	F (1, 28) = 0.2698, P=0.6076	F (1, 28) = 0.02393, P=0.8782
Interleukin 13	IL-13	F (1, 28) = 1.053, P=0.3135	F (1, 28) = 2.703, P=0.1114	F (1, 28) = 0.2256, P=0.6385
Interleukin 15	IL-15	F (1, 28) = 0.03056, P=0.8625	F (1, 28) = 0.01608, P=0.9000	F (1, 28) = 1.850, P=0.1847
Keratinocyte Chemoattractant, also called chemokine ligand 1 (CXCL1)	KC/CXCL1	F (1, 28) = 0.02370, P=0.8788	F (1, 28) = 1.830, P=0.1869	F (1, 28) = 0.1300, P=0.7211
CXC Chemokine ligand 5 (CSCL5)	LIX/CSCL5	F (1, 28) = 3.633, P=0.0670	F (1, 28) = 3.716, P=0.0641	F (1, 28) = 0.7534, P=0.3928
Macrophage colony stimulating factor	M-CSF	F (1, 28) = 0.001212, P=0.9725	F (1, 28) = 1.624, P=0.2130	F (1, 28) = 0.4762, P=0.4958
Monocyte chemoattractant protein-1	MCP-1	F (1, 28) = 1.282, P=0.2671	F (1, 28) = 0.5134, P=0.4796	F (1, 28) = 0.2248, P=0.6391
Chemokine ligand 9 (CXCL9)	MIG/CXCL9	F (1, 28) = 2.252, P=0.1446	F (1, 28) = 1.453, P=0.2382	F (1, 28) = 0.2647, P=0.6109
Macrophage inflammatory protein-1 alpha	MIP-1α	F (1, 28) = 0.01615, P=0.8998	F (1, 28) = 0.7459, P=0.3951	F (1, 28) = 0.2446, P=0.6247
Macrophage inflammatory protein-1 beta	MIP-1β	F (1, 28) = 0.5789, P=0.4531	F (1, 28) = 0.4847, P=0.4920	F (1, 28) = 0.08603, P=0.7714
Macrophage inflammatory protein-2	MIP-2	F (1, 28) = 1.258, P=0.2716	F (1, 28) = 0.2087, P=0.6513	F (1, 28) = 0.08053, P=0.7787
Regulated upon activation, normal T cell expressed and presumably secreted	RANTES/CCL5	F (1, 27) = 3.896, P=0.0587	F (1, 27) = 0.3129, P=0.5805	F (1, 27) = 0.2819, P=0.5998
Tumor necrosis factor alpha	TNFα	F (1, 28) = 1.32, P=0.2588	F (1, 28) = 0.6525, P=0.4260	F (1, 28) = 0.1174, P=0.7345

Supplemental Table 1. Results of the Mouse Cytokine Discovery Assay. The p values reported in this table did not survive false discovery rate corrections.

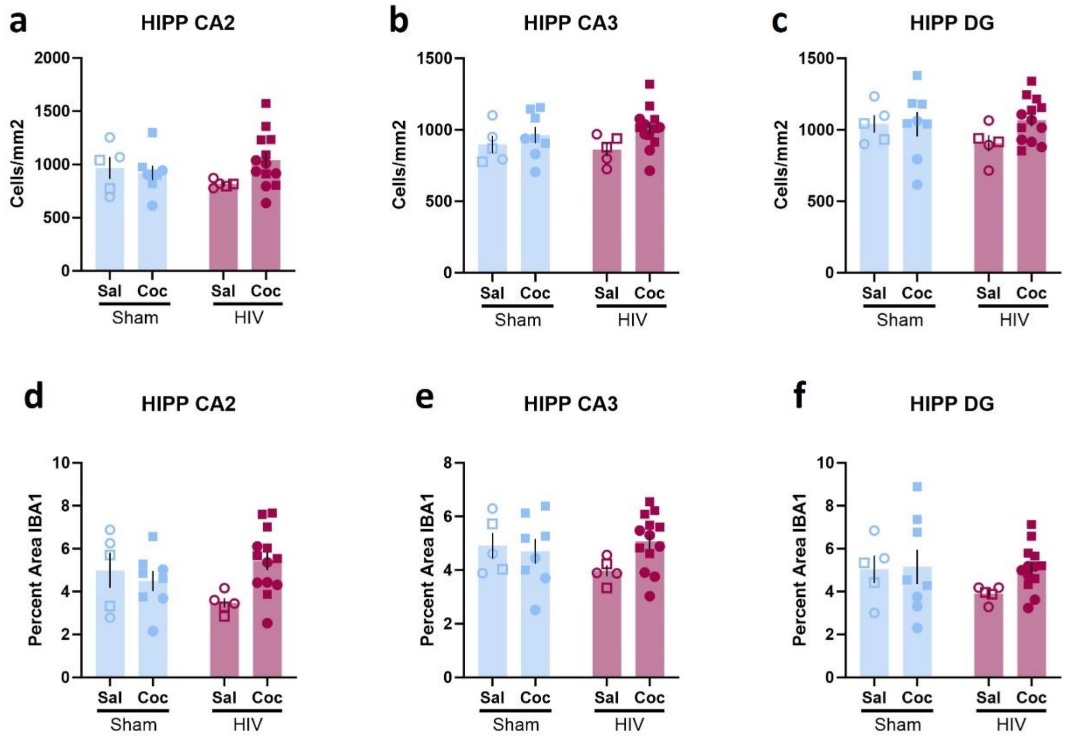

Supplemental Figure 1. IBA1 cells per mm2 in the (a) CA2, (b) CA3, and (c) DG of the hippocampus. Percent area of IBA1 in the (d) CA2, (e) CA3, and (f) DG of the hippocampus.
