# Supplementary material for: Impaired extinction of cocaine seeking in HIV-infected mice is accompanied by peripheral and central immune dysregulation
Source: Commun Biol. 2024 Mar 30;7:387. doi: 10.1038/s42003-024-06079-8 (PMC10980811; doi:10.1038/s42003-024-06079-8)
Supplement: Supplementary file 3 — Description of Supplementary Materials [file 42003_2024_6079_MOESM3_ESM.docx]

**Description of Additional Supplementary Files**

**File name:** Supplementary Data 1

**Description:** All analyzed data contained in this manuscript
