# Supplementary material for: Impaired extinction of cocaine seeking in HIV-infected mice is accompanied by peripheral and central immune dysregulation
Source: Commun Biol. 2024 Mar 30;7:387. doi: 10.1038/s42003-024-06079-8 (PMC10980811; doi:10.1038/s42003-024-06079-8)
Supplement: Supplementary file 5 — Reporting Summary [file 42003_2024_6079_MOESM5_ESM.pdf]

nature portfolio

Corresponding author(s): Jacqueline Barker

Last updated by author(s): Mar 19, 2024

Reporting Summary

Nature Portfolio wishes to improve the reproducibility of the work that we publish. This form provides structure for consistency and transparency in reporting. For further information on Nature Portfolio policies, see our Editorial Policies and the Editorial Policy Checklist.

Statistics

For all statistical analyses, confirm that the following items are present in the figure legend, table legend, main text, or Methods section.

n/a	Confirmed	
☐	☒	The exact sample size (*n*) for each experimental group/condition, given as a discrete number and unit of measurement
☐	☒	A statement on whether measurements were taken from distinct samples or whether the same sample was measured repeatedly
☐	☒	The statistical test(s) used AND whether they are one- or two-sided<br>*Only common tests should be described solely by name; describe more complex techniques in the Methods section.*
☒	☐	A description of all covariates tested
☐	☒	A description of any assumptions or corrections, such as tests of normality and adjustment for multiple comparisons
☐	☒	A full description of the statistical parameters including central tendency (e.g. means) or other basic estimates (e.g. regression coefficient) AND variation (e.g. standard deviation) or associated estimates of uncertainty (e.g. confidence intervals)
☐	☒	For null hypothesis testing, the test statistic (e.g. *F*, *t*, *r*) with confidence intervals, effect sizes, degrees of freedom and *P* value noted<br>*Give P values as exact values whenever suitable.*
☒	☐	For Bayesian analysis, information on the choice of priors and Markov chain Monte Carlo settings
☒	☐	For hierarchical and complex designs, identification of the appropriate level for tests and full reporting of outcomes
☐	☒	Estimates of effect sizes (e.g. Cohen's *d*, Pearson's *r*), indicating how they were calculated

Our web collection on statistics for biologists contains articles on many of the points above.

Software and code

Policy information about availability of computer code

Data collection: Behavioral data was collected using Med-Associates software as indicated.

Data analysis: Analysis was performed using GraphPad Prism.

For manuscripts utilizing custom algorithms or software that are central to the research but not yet described in published literature, software must be made available to editors and reviewers. We strongly encourage code deposition in a community repository (e.g. GitHub). See the Nature Portfolio guidelines for submitting code & software for further information.

Data

Policy information about availability of data

All manuscripts must include a data availability statement. This statement should provide the following information, where applicable:

- Accession codes, unique identifiers, or web links for publicly available datasets
- A description of any restrictions on data availability
- For clinical datasets or third party data, please ensure that the statement adheres to our policy

Source data for all figures are provided in the supplemental data.

Research involving human participants, their data, or biological material

Policy information about studies with human participants or human data. See also policy information about sex, gender (identity/presentation), and sexual orientation and race, ethnicity and racism.

Reporting on sex and gender	n/a
Reporting on race, ethnicity, or other socially relevant groupings	n/a
Population characteristics	n/a
Recruitment	n/a
Ethics oversight	n/a

Note that full information on the approval of the study protocol must also be provided in the manuscript.

Field-specific reporting

Please select the one below that is the best fit for your research. If you are not sure, read the appropriate sections before making your selection.

☒ Life sciences ☐ Behavioural & social sciences ☐ Ecological, evolutionary & environmental sciences

For a reference copy of the document with all sections, see nature.com/documents/nr-reporting-summary-flat.pdf

Life sciences study design

All studies must disclose on these points even when the disclosure is negative.

Sample size	Sample size was determined by power analysis and precedent in the literature.
Data exclusions	Data from 1 mouse was excluded from the cocaine-paired chamber latency analysis (Fig 4C) as a statistical outlier.
Replication	The experimental findings were successfully reproduced in 3 cohorts of mice over 3 years.
Randomization	Mice were matched for HIV infection or sham-infection based on engraftment data and sex, in order to have representation among all groups. For conditioned place preference tests, mice were matched for cocaine or saline-only based on initial preference data in order to prevent initial biases from impacting results.
Blinding	Investigators were blind to experimental groups during data analysis. Investigators were not blind during data collection due to the potentially hazardous nature of these mice.

Reporting for specific materials, systems and methods

We require information from authors about some types of materials, experimental systems and methods used in many studies. Here, indicate whether each material, system or method listed is relevant to your study. If you are not sure if a list item applies to your research, read the appropriate section before selecting a response.

Materials & experimental systems

n/a	Involved in the study	
☐	☒	Antibodies
☒	☐	Eukaryotic cell lines
☒	☐	Palaeontology and archaeology
☐	☒	Animals and other organisms
☒	☐	Clinical data
☒	☐	Dual use research of concern
☒	☐	Plants

Methods

n/a	Involved in the study	
☒	☐	ChIP-seq
☒	☐	Flow cytometry
☒	☐	MRI-based neuroimaging

Antibodies

Antibodies used	Anti-Glial Fibrillary Protein antibody produced in rabbit (Sigma-Aldrich, G9269), Anti Iba1, Rabbit (FUJIFILM Wako Pure Chemical

Antibodies used	Corporation, 019-19741), Biotin-SP (long spacer) AffiniPure Donkey Anti-Rabiit IgG (H+L) (Jackson ImmunoResearch Laboratories Inc., 711-065-152).
Validation	Sigma Aldrich states, "Our standard antibody validation processes include verification for each recommended immunodetection application. Each of the thousands of antibodies in our portfolio are certified through our standard validation process to ensure quality and reproducibility."

Animals and other research organisms

Policy information about studies involving animals; ARRIVE guidelines recommended for reporting animal research, and Sex and Gender in Research

Laboratory animals	Adult male and female NOD/Scid IL2Rg-/- (NSG) mice were used for these studies. Average age upon arrival was 26 weeks.
Wild animals	This study did not involve wild animals.
Reporting on sex	25 male and 40 female mice were utilized in this study.
Field-collected samples	This study did not involved samples collected from the field.
Ethics oversight	All protocols involving the use of animals in this study were approved by the Drexel University Institutional Animal Care and Use Committee (IACUC).

Note that full information on the approval of the study protocol must also be provided in the manuscript.
